# Impact of Whole Body Electromyostimulation on Velocity, Power and Body Composition in Postmenopausal Women: A Randomized Controlled Trial

**DOI:** 10.3390/ijerph17144982

**Published:** 2020-07-10

**Authors:** Alvaro Pano-Rodriguez, Jose Vicente Beltran-Garrido, Vicenç Hernandez-Gonzalez, Natalia Nasarre-Nacenta, Joaquin Reverter-Masia

**Affiliations:** 1 Research Group Human Movement, University of Lleida, E-25001 Lleida, Spain; 2 EUSES Escola Universitària de la Salut i l’Esport, Rovira i Virgili University, 43870 Tarragona, Spain; jose.vicente@euseste.es (J.V.B.-G.); reverter@didesp.udl.cat (J.R.-M.); 3 Nutritional Coaching SL, 08011 Barcelona, Spain; vicenc.hernandez@udl.cat (V.H.-G.); natalianasarre@gmail.com (N.N.-N.)

**Keywords:** whole-body electrical muscle stimulation, whole-body electrostimulation, physical exercise, aging, public health

## Abstract

Menopause is associated with losses in strength and power along with weight and fat mass gains, which may result from menopause-related hormonal changes, aging-associated diseases, and decreased physical activity time. The objective of this study is to analyze if whole-body electromyostimulation (WB-EMS) is suitable for the prevention and treatment of postmenopausal physical deterioration. Thirty-four healthy sedentary women between 55 and 69 years followed an experimental design pre-post test. Both groups conducted 10 weeks of aerobic and strength training program. The experimental group conducted the training with superimposed WB-EMS during exercise. At the end of the intervention, the experimental group obtained better power (Squat: mean difference (MD) = 38.69 W [1.75,75.62], *d* = 0.81; Bench press: MD = 25.64 W [17.48, 33.82], *d* = 2.39) and velocity (Squat: MD = 0.04 m·s^−1^ [0.01, 0.08], *d* = 0.98; Bench press: MD = 0.10 m·s^−1^ [0.06, 0.14], *d* = 1.90) score improvements than the other group (*p_Bonferroni_* < 0.05). Furthermore, trivial to small effects were found in the body composition of the participants of both groups (*p* > 0.050). WB-EMS showed a favorable isolated effect on the development of power and velocity, but it induced negligible effects on the body composition of postmenopausal women.

## 1. Introduction

Aging is associated with a decline of functional capacity, which damages the quality of life and increases the level of elder’s dependence. [1] As a result, there is global concern nowadays, both for what aging means for the health of the elders and the increase in public spending associated with it [2]. Conceptually, functional capacity represents the physical capability that is needed to undertake usual everyday activities, independently and without the early onset of fatigue [3]. One of the factors that affect negatively on physical capacity is the progressive loss of skeletal muscle mass and strength, which is known as sarcopenia. [4,5] Thus, it is established that reduced muscle strength with aging leads to the loss of functional capacity and is a major cause of disability, mortality, and other adverse health outcomes [6].

While the mechanisms by which these functional limitations occur are multifactorial and unclear, changes in body composition, such as losses in muscle mass coupled with increases in fat mass (obesity) have been identified as significant contributors [7]. Special attention should be given to the population of postmenopausal women, who are characterized by the highest percentage of body fat (PBF) and by the lowest contents of lean tissue in the body [8]. Thus, interventions known to combat these deleterious changes are imperative.

Low physical activity is associated with frailty phenotype, which includes unintentional weight loss, self-reported exhaustion, weakness, and slow walking speed [9]. It leads to conclude that sedentary behavior is extremely considered as a trigger factor of the dependency due to the deterioration of the elderly’s health that it entails. It has been reported that inactive healthy older adults had two times higher mortality risk compared with same-age physically active older adults [10]. Despite all the benefits it offers as compensation for the deterioration of physical condition and health, physical activity has been a resource in critical disuse as a preventive strategy for healthy aging [11]. Mainly, sedentary behavior can be observed as age progresses [12]. Therefore, to overcome a sedentary lifestyle and prevent the deterioration of strength and body composition in postmenopausal women, it will be necessary to generate physical activity programs with an attractive profile. These programs should provide easily perceived benefits and should enjoy good social support [13].

The whole-body electromyostimulation (WB-EMS) consists of the application of a biphasic and symmetrical current using a specific suit connected to an electrostimulation device. The devices generally allow the activation of the thighs, arms, buttocks, abdomen, chest, lower back, upper back, wide dorsal, and with two auxiliary channels of free choice. In recent years, high-intensity training programs have been developed with older adults, observing increases in strength [14,15,16,17,18] and body composition [19,20]. Taking into account that the WB-EMS can become easily intense and guarantees sufficient effort in those unable or reluctant to do it on their own initiative [21], we hypothesize that it could be an appropriate training methodology for sedentary postmenopausal women. Kemmler et al. [22] compared the effects of a WB-EMS training with those of a traditional high-intensity interval training, concluding that both programs showed the same effectiveness in improving the physical condition of sedentary men with cardio-metabolic risk. Other studies have analyzed the effects of WB-EMS on the health of older people, observing improvements in sarcopenia [21,23,24,25,26,27,28] and body composition [23,29]. In addition, the WB-EMS has established itself as an effective method of physical conditioning, achieving improvements in maximum isometric strength of the leg extenders, vertical jump, and handgrip strength [21,22,28,30,31].

Despite the optimistic results that the scientific literature shows, a recent systematic review concluded that, at present, there is little evidence regarding the effectiveness of WB-EMS focusing on the improvement of power, velocity, and body composition in the elderly [32]. On the topic of this controversy, new studies whose protocols adequately conform to the scientific methodology should be carried out. Thus, the objective of this study is to analyze, from a broad and realistic perspective, the influence of a 10-week WB-EMS training program on the power, velocity, and body composition of postmenopausal women.

## 2. Materials and Methods

### 2.1. Experimental Approach

The experimental procedure of the study corresponded to a two-arm randomized trial with parallel-groups. There were no changes in the protocol since the start of the study. The reporting has been done following the Consolidated Standards of Reporting Trials (CONSORT) guidelines for standard items in interventional trials [33].

This study is part of a large project conducted from September to November 2018, and data related to Physical fitness after 10 weeks of WB-EMS training (i.e., balance, strength, flexibility, agility, speed, and cardiovascular resistance) have been published elsewhere [34]. In the present manuscript, we included the comparisons between pre to post 10 weeks focused on power and velocity as well as body composition. This study received ethical approval from the committee of Arnau of Vilanova’s University Hospital, Lérida (Spain), and was conducted in accordance with the Declaration of Helsinki. Trial registration: ISRCTN15558857 last edited: 02/12/2019 (retrospectively registered).

### 2.2. Participants

Thirty-four postmenopausal women living in Lleida (Spain) were recruited to participate in this investigation voluntarily. The recruitment and follow up period elapsed from June 2018 to April 2019. Briefly, in a first step, they were contacted by a phone call to be informed about the nature of the project. All of them were invited to attend an informational meeting where more details were given on the benefits and possible risks that their participation in the project might entail. Those who showed interest in their participation were recruited according to the inclusion criteria. The inclusion criteria were as follows: (1) no reported contraindications (i.e., total endoprosthesis, abdomen/groin hernia, epilepsy, and cardiac arrhythmia) for WB-EMS intervention, (2) sedentary status according to the scales provided by the Eurobarometer (below 600 MET-minute per week) [35], (3) postmenopausal status (detailed below in a separated section). Written informed consent was obtained from the whole sample. Participants were allocated and informed about their assigned arm by a phone call, which was made by an external collaborator. They were also assured of their anonymity and the reporting of their views in aggregate form to protect their identities.

Menopause status: Hormone assessments were performed from fasting serum samples taken between 8:00 and 10:00 AM. The serum was separated by centrifugation for 10 min at 2200× *g*. Systemic FSH levels were immunoassayed using IMMULITE 2000 XPi (Siemens Healthcare Diagnostics, Frimley, Camberley, UK). The participants’ menopause status was determined based on the self-reported menstrual cycle.

Applying the categorization of Kovanen et al. [36], subjects were postmenopausal if no menstrual bleeding during the past six months and following cut values were applied FSH > 30 IU/L. Participants self-reported their health problems, gynecologic status, and use of medications.

### 2.3. Interventions

Two familiarization sessions for testing and training took place one week before the pre-test. The training familiarization consisted of two sessions performed at a maximum of 12 min with low/submaximal intensity, as it was previously recommended [37]. After, during the 10-week training period, participants performed 20 training sessions (TS; 2/week) with a minimum of 48 h of rest between sessions. The training program was the same for both groups, but the 1st group conducted the training exercises with superimposed WB-EMS (EX + WB-EMS), and the 2nd group performed the training exercises without superimposed WB-EMS (EX). Groups conducted their training sessions separately on different weekdays, and participants from one group did not know the existence of the other group. They were asked not to make physical efforts outside the intervention.

The training program consisted of two resistance blocks. The participants had to perform in each block 20 repetitions (6 s for two rep and then 4 s rest) in three exercises (squat, deadlift, and bench press) recommended for older people [38]. The additional load for every participant was adjusted to 40% repetition maximum (RM) obtained by an indirect measurement test [39]. After strength exercises, participants performed a 10-min cardiovascular workout on the treadmill, at a constant individualized speed, obtained from the talk test [40]. The highest speed they could walk while talking was estimated. As it was done in Wirtz et al. [41], the absolute load in resistance training and speed in cardiovascular training was increased by 5% every two weeks to apply the principle of progressive overload. As a cooldown at the end of the sessions, 10 min of stretching exercises were done. In the whole sample, the assessment of the exertion perceived was controlled at the end of the training sessions with a 20-Borg scale. The intention was always not to exceed the level 15 (“Hard”) [42].

**Figure 1:**
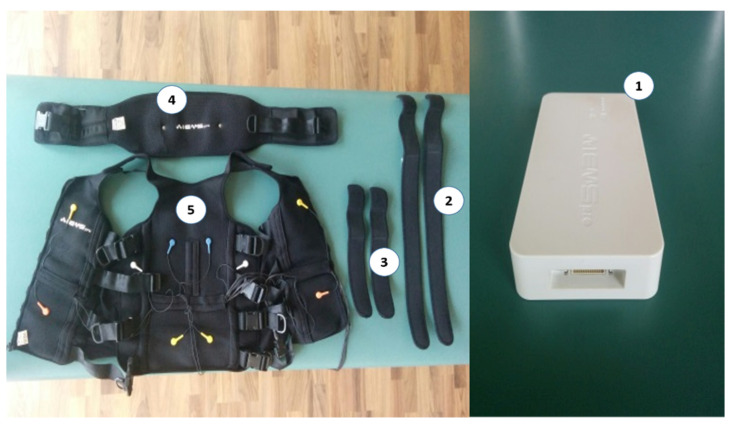
Wiemspro equipment. (1) The electromyostimulator device, (2) Strap electrodes for the thighs, (3) Strap electrodes for the arms, (4) Belt with electrodes for the buttocks, (5) Vest with electrodes for the abdomen, chest and back area.

The electrostimulation (EMS) surface electrodes (Wiemspro^®^ electrostimulator, Malaga, Spain) [43] were applied in the whole body matching the electrical stimulus with the repetitions. The complete electrostimulation equipment, consisting of the suit and the electrostimulator device, does not weigh more than 1.5 kg (see Figure 1).

Given that subcutaneous fat modifies the transmission of the electrical stimuli into muscle [44], the current intensity had to be normalized in the WB-EMS training. Following a procedure similar to that of Kemmler et al. [23], the intensity of the applied electrical stimulus was matched to the 6–8 level of intensity perception of the electrical current (IPC) on a 0 to 10 pain scale. This represented an intensity that enabled the dynamic movement as pre-testing in the laboratory [45].

During the strength exercises, a frequency of 55 Hz was applied, since it was observed previously that a current frequency of ≥50 Hz is adequate for the activation of type II fibers, and therefore, cause adaptations in the strength training [46]. Pulse width: leg and glute 350 µs, lumbar, rectus abdominis and latissimus dorsi 300 µs, trapezius 250 µs, chest 200 µs, and arms 150 µs. Eight hundred ms of ascent ramp and descent ramp of 500 ms [47,48], with a 60% duty cycle were used [46]. Taking into account the effectiveness of low-frequencies of electrostimulation on the aerobic capacity [49], during cardiovascular training on the treadmill, the current applied was 7 Hz (ratio of on-time to the total cycle time: % duty cycle = 100/[total time/on-time]), considering previous methodological issues in low-frequencies [50]. The training protocols were supervised by two instructors who graduated in physical activity and sports sciences with wide experience in WB-EMS training.

Harms: Adverse events, including physical injuries, were monitored by the instructors of the intervention and the responsible staff of the assessments and documented through the facility’s incident reporting process.

### 2.4. Outcomes

#### 2.4.1. Primary Outcomes: Body Composition

As primary outcomes, the following parameters were measured: height, weight, body mass index (BMI), body fat percentage, fat mass, visceral fat, lean mass, abdominal fold, contracted arm perimeter, waist perimeter, hip perimeter, and the sum of six-folds.

Height was determined with an accuracy of 0.10 cm with a stadiometer (SECA, Hamburg, Germany). The participants were standing erect without shoes, with heels together and their heads in the Frankfort horizontal plane. The bodyweight was evaluated with an electronic balance with a sensitivity of 0.10 kg (Tanita BC-418 MA, Tanita Corp. Tokyo, Japan). The body mass index was obtained using the formula: bodyweight/height^2^. The fat mass, visceral fat and lean mass were estimated by bioelectrical impedance analysis (BIA) using an eight-contact electrode segmental body composition analyzer (Tanita BC-418 MA, Tanita Corp. Tokyo, Japan).

To assess skinfolds and perimeters, a 0.50 mm sensitivity Slim Guide caliper and a measuring tape (CESCORF) were used, respectively [51]. The muscle mass was estimated by using the formula of Lee et al. [52].

All measurements were made in duplicate non-consecutively and using the average value as the final value. All women were measured at the same time of the day for baseline and post-test, and they were instructed to avoid alcohol consumption and maintain the usual habits in fluid/food intake to avoid errors due to differences in hydration. All analyzes were performed by a level I anthropometric technician certified by the International Society for the Advancement of Kinanthropometry (ISAK), as described in its reference manual [53].

#### 2.4.2. Secondary Outcomes: Power and Velocity

For the secondary outcomes, a progressive resistance test (PRT) [54] was carried out to make an accurate assessment of strength development. The PRT facilitates simultaneous direct calculations of velocity (m·s^−1^) and power (W), produced with different loads, and at the same time. Taking into account that as a consequence of aging, the losses in strength are observed mostly in the type II fibers [55], the abovementioned variables were the primary outcomes in this study.

Following the PRT test protocol, we assessed the execution of six to eight series of two to three repetitions in squat and bench press, applying the maximum possible acceleration alternated with rest intervals of 2 to 5 min. The rest period was proportional to the intensity and duration of the effort to avoid the prediction errors caused by the accumulated fatigue. The load was increased progressively with the sets. For each magnitude of weight lifting, it is necessary to select the repetition with which the highest values of average velocity and power are reached, as this factor expresses the highest mechanical efficiency of the exercise [56]. In this study, the best repetition of the best set was recorded. Then, the load in which that best set was done in the baseline, was used in the post-test to compare the evolution of the variables after the intervention in the post-test.

To perform this test, a lineal encoder Chronojump^®^ (BoscoSystem, Barcelona, Spain) was used to detect the position of the weight bar during linear movements. This device warranties the viability and reliability of data, offering an accurate estimation of the range of movement, acceleration, velocity, strength and the power produced during each action [57].

### 2.5. Sample Size

The minimum sample size needed was determined by an a-priori power analysis using the G*Power3 software (University of Duesseldorf, Duesseldorf, Germany) for Mac [58] following the indications of Beck [59]. The effect size value used was: *d* = 0.70, with a total sample size of *n* = 30, based on a previous study in postmenopausal women [23]. Furthermore, two levels for the between-subject factor (EX + WB-EMS, EX), two levels for the within-subject factor (Baseline, postintervention), alpha error probability set at 0.05 and a power of 0.80 were used. This analysis indicated a minimum total sample size of 20 participants. Considering a dropout rate of 25%, 13 participants per arm were required. In order to check the minimum effect size to which the model was sensitive, a sensitivity analysis with the overestimated sample size assuming a 25% dropout rate (*n* = 26) was done. The power and alpha values used were 0.80 and 0.05, respectively. The model was sensitive enough to detect effects as small as *d* = 0.57.

### 2.6. Randomization

**Figure 2:**
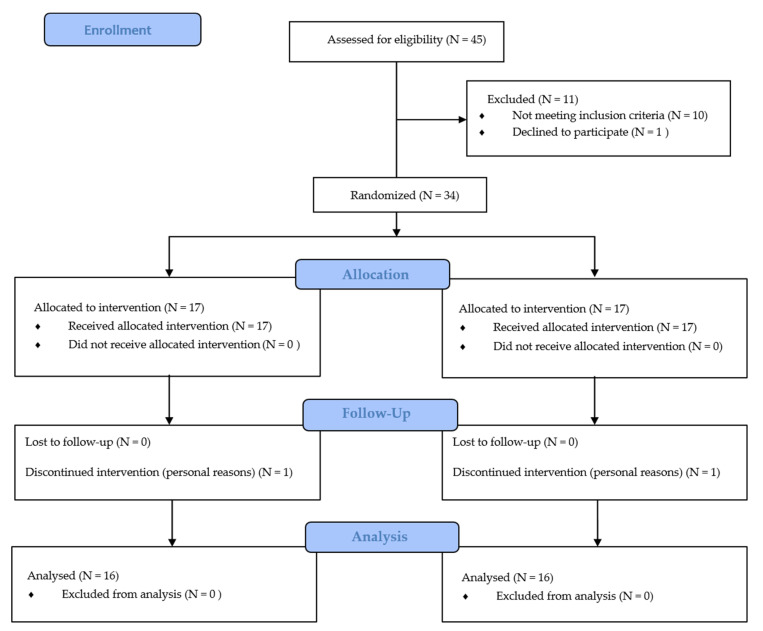
Consolidated Standards of Reporting Trials (CONSORT) flow diagram. This figure shows the flow of participants through the trial according to the criteria recommended in the CONSORT guidelines.; EX = Voluntary exercise group; EX + WB-EMS = Voluntary exercise with whole-body electromyostiulation (WB-EMS).

The randomization of the study sample was carried out by a computer random number generator [60]. The participants were randomized into two different groups by simple randomization. The 1st group conducted a voluntary exercise program with superimposed WB-EMS (EX + WB-EMS, *n* = 17), and the 2nd group performed only voluntary exercise training (EX, *n* = 17) (See Figure 2). The assessments were carried out in the sports center Ekke, located in Lleida (Spain). The data were recorded by blinded testers in a spreadsheet that was stored in an encrypted USB memory by an external collaborator, to guarantee the privacy of the participants. The collaborator assessed the safety and validity of the research data. A blinded statistician had access to the final dataset of the study. The participants were asked not to take any stimulants before assessments to avoid their influence on the results.

### 2.7. Statistical Procedures

All the participants who started the intervention were included in the statistical analyses. Data are presented in mean ± standard deviation (SD). The assumption of normality was verified by exploring the Q-Q plots and histogram of residuals. The homogeneity assumption was checked using the Levene’s test.

To assess between groups’ sample characteristics differences at baseline, an independent sample t-test was performed. If groups differed in any sample characteristics, it was used as a covariate.

To detect between-groups’ effectiveness differences, analysis of covariance, using the baseline values as a covariate, was used [61,62,63]. When significant F values were found, post-hoc test with Bonferroni correction was applied.

Within-group changes were assessed with an ANOVA procedure and post-hoc tests with the Bonferroni correction.

The Cohen’s *d* effect sizes (ES) were reported with 95% confidence intervals (CI) and interpreted as: <0.2 = trivial; 0.2–0.6 = small; 0.6–1.2 = moderate; 1.2–2.0 = large; >2.0 = very large [61].

The significance level was set at α = 0.05 for all test. All statistical analyses were performed using JASP (version 0.12.2; JASP Team (2019), University of Amsterdam, Amsterdam, The Netherlands).

## 3. Results

At the start of the study, 17 participants were randomly assigned to each intervention group. One participant of each group left the study due to personal reasons without attending to the baseline assessments. Finally, 16 participants in each group were fully assessed, received the intended interventions, and were analyzed for both the primary and secondary outcomes.

The percentage of attendance of both groups was higher than 85% (EX + WB-EMS: 92.76 ± 6.34% vs. EX: 89.47 ± 9.80%) and the perceived exertion was “Somewhat hard” (13 to 14) in the 20-Borg scale (EX + WB-EMS: 14.15 ± 0.65 arbitrary units (AU) vs. EX: 13.21 ± 0.91 AU).

### 3.1. Sample Characteristics

The baseline characteristics of the study sample are shown in Table 1. A statistically significant mean difference in age was shown between groups (*MD* [95% CI] = 3.35 years [0.82, 5.89], *p* = 0.011).

### 3.2. Body Composition

A post-hoc analysis within (time) on the body composition variables for both groups are reported in Table 2. None of the groups obtained statistically significant improvements in any of the variables, with magnitudes of effects sizes ranging from trivial to small.

Analysis examining differences in the improvements of body composition at the end of follow-up among EX + WB-EMS versus EX groups, adjusting by the age and the corresponding value scores at baseline, are displayed in Table S1 of the Supplementary Materials.

### 3.3. Power and Velocity

The post-hoc analysis within (time) on the mechanical variables of strength exercises for both groups are reported in Table 3. The EX + WB-EMS group obtained statistically significant improvements in all variables with very large effect sizes. However, the EX group obtained statistically significant improvements with large effect sizes in the mechanical variables of the squat exercise but not in the bench press exercise (trivial effect sizes).

The analysis examining the differences in the improvements of mechanical variables of the strength exercises among EX + WB-EMS versus EX groups, adjusting by age and the corresponding value score at baseline, are displayed in Table 4. At the post-test, the participants in the EX + WB-EMS group showed better velocity and power score improvements in both exercises than their peers in the EX group with effect sizes ranging from moderate to very large (*p* < 0.05). See Figure S1 of Supplementary Materials.

## 4. Discussion

The overarching aim of the present trial was to determine the effect of a WB-EMS training program on postmenopausal women, focusing on improving both body composition and low and high extremity power through resistance training and cardiorespiratory exercises. The main findings were that voluntary exercise with WB-EMS promoted higher increases in power and velocity on squat and bench press than voluntary exercise alone. On body composition, negligible effects were found in both groups.

### 4.1. Body Composition

Aging is associated with various modifications in body composition, including changes in weight, loss of muscle mass, and an increase in fat mass. This is mostly observed in postmenopausal women, who present the highest percentage of body fat and the lowest lean body mass, soft tissue percentage, and total body water due to a decline in endogenous estrogen production [8,64]. As frailty, dependence, and fall risk are characterized exactly by the mentioned modifications [65]. Thus, some solutions should be proposed from the scientific circles to reduce the impact of aging on this population and guarantee a better quality of life.

The results of this study showed no changes in body composition after 10 weeks of an EB-EMS training program. Some previous studies assessed the effects of WB-EMS on the body composition of populations in advanced age. Kemmler et al. (2010) [23] observed improvements in variables like bodyweight and total abdominal fatness of postmenopausal women, but the authors indicate that there may be a synergistic effect that favored the results of the WB-EMS group. The same research group found no statistical improvements in muscle mass of untrained old adult men [66], which is in accordance with the results of this study. They also found a better improvement in fat mass but not in bodyweight, comparing the WB-EMS group with a stretching training group. In a study called Test III trial, Kemmler et al. [21,29] and Stengel et al. (2015) [24] did not find improvements in bodyweight, total fat mass, or bone mineral density, but they did to some extent and with high variability in total lean mass and body fat. In another study [25,67], the authors only observed differences in muscle mass comparing a WB-EMS group with a control group who only performed slight movements in a supine position; what is not clear is the analysis of the isolated effect of WB-EMS. Optimistic results could be observed in a recent study made with community-dwelling older men [27,28,68], where improvements in body composition were found, but their control group did not carry out any kind of training, just as in Schink et al. [69]. Studies carried out with younger populations to assess the effects of WB-EMS on body composition did not observe statistical differences [70,71,72,73]. All those studies were carried out during week-long experimental phases. So, the present study found similar results. Thus, future studies with longer interventions are necessary to assess the possible effects on body composition after more extended WB-EMS exposure periods.

The unchanged lean mass after our experimental phase should imply no changes in muscle mass, which could be contradictory with the improvements found in power and velocity. These improvements could be explained by the nervous system adaptations discussed above. Instead of increments in muscle mass, the beneficial effects would come from neural efficiency enhancements, which would develop the capacity of the motor unit recruitment.

Kemmler et al. [74] assessed energy expenditure in both voluntary or WB-EMS training. Unlike the present study, the sample was composed of young males, but it is interesting to point out that the authors found a relatively small isolated effects of WB-EMS exposure. This little influence of WB-EMS on energy expenditure could be an explanation of the unchanged results. However, future studies should consider dietetic control to assess in a more precise way, the caloric intake-outtake balance in the context of WB-EMS exposure.

### 4.2. Power and Velocity

Decreases in muscle function commonly observed with aging are greatly related to impairments in muscle strength and power [1,75]. In this respect, strategies aiming to increase muscle strength and power to preserve functionality in the elderly are of interest.

The results observed in the present study show that WB-EMS triggers interesting improvements in power and velocity in postmenopausal women. These findings are in concordance with some previous studies observing that WB-EMS increases the maximum dynamic and isometric strength in older individuals [21,23,24,28,29]. It must be pointed that the study cohort is, on average, 10 years younger than the mentioned previous studies, but we consider mentioning them as a probe to show that WB-EMS might seem to be a suitable method to improve the explosive strength component despite the age component [31].

However, another study observed different results than ours. Amaro-Gahete et al. [76] carried out a 12-week randomized controlled trial with a parallel-group in which one of the groups performed a high-intensity interval training (HIIT group), and the other group performed a training program “with similar characteristics to those used for the HIIT group adding whole-body electromyostimulation”. Unfortunately, the authors did not observe better evolution of the strength variables in the WB-EMS group than in the HIIT group. This controversy on the strength evolution in advanced age people shows the need for future clinical trials approaching this research line.

Several reports have highlighted a greater age-related decline in lower limb explosive capacity compared with maximal muscle strength [77,78,79,80]. One of the most important declines coming from aging is the manifest at the muscle fiber level by type II atrophy [81], which is accompanied by a specific decline of this type of fiber in skeletal muscle stems or satellite cell number and function [82,83]. All these processes have, as a consequence, a reduction of the movements velocity and power in the elderly. The decline in explosive capacity in older adults has been linked to impaired ability to perform daily living tasks, including climbing stairs and rising from a chair together with reduced ability to recover from a trip or a slip, which is important in fall prevention and independence [84]. Due to this, it is of extreme importance the training and assessment of these variables in older populations. Kemmler et al. [23] found a significant increase in the isometric leg power of elderly males. Wirtz et al. [72] found improvements in the leg flexors in an isoinertial power test with a leg curl machine. The same happened in the leg curl power strength in Wirtz et al. [85]. Their studies were carried out with young trained males, but they agreed with the results of the present study in the successfulness of the WB-EMS enhancing the power in an isolated way.

It must be pointed out that most of the previous studies implemented WB-EMS 1 to a maximum of 1.5 sessions a week. In the present study, the exhaustive control of the training loads allowed two weekly training sessions. It was done by monitoring the current intensity using the abovementioned IPC pain scale during the WB-EMS + EX group training, along with the assessment of the exertion perceived with 20-Borg scale at the end of both group sessions.

Most of the previous studies used 85 Hz while the present used 55 Hz. Our results confirmed the previous evidence that a current frequency of ≥50 Hz is adequate for the activation of type II fibers, and therefore, for cause for the adaptation to strength training [46]. While the general pulse wide of 350 µ was found in some of the other studies, we decided to use a more specific pulse width for each muscle group to guarantee the maximum comfort of the trainee as it was previously proceeded in Amaro-Gahete [76].

To the best of the author’s knowledge, the present study is the first to assess the effects of WB-EMS on power and velocity in postmenopausal woman using a lineal encoder, what guarantees the reliability and precision of the data.

An explanation of the observed results in this study could be the preferential adaptations of the type II fibers as a consequence of the application of electrical current, as it was observed in previous studies with local EMS [86,87], but it is not clear, taking into account that in those studies the control groups did not proceed to a comparable isometric training, so the analysis of EMS by its own becomes confusing. The evolution of type II fibers after a WB-EMS training is an interesting research line still in the early steps, given that only one previous study has been published discussing it [73]. The authors did not observe statistical differences between the WB-EMS group and the training group.

A further explanation of the results could be due to neural factors acting at various levels of the nervous system, which could result in increasing the maximal level of muscle activation [86,87,88,89]. As it is established, recruitment patterns during electrical stimulation are random. Both slow and fast fibers are activated non-selectively [90]. Small diameter axons close to the electrode could depolarize with lower stimulus amplitudes than larger axons further away, resulting in random motor unit recruitment orders of specific types [91]. Consequently, conventional electrical current recruits fewer fatigue-resistant motor units, and more fast-fatigable ones compared with voluntary contractions. Thus, the electrical current could have improved the ability to activate fast fibers that would not typically be recruited during normal daily activities [92]. This may be the mechanism primarily responsible for the power and velocity gains.

Study Limitations: Although this study has remarkable strengths, such as (1) the precise analysis of power and velocity with a linear encoder, (2) the comparable treatments in both the WB-EMS and the control group, and finally, (3) the high ratio of the sample participation during the sessions (>85%); certain limitations could be taken into consideration. First, nutritional control of the sample was not carried out throughout the treatment, since only instructions regarding keeping on with the usual diet were given to participants. The importance of caloric intake control is necessary for the aiming of body composition improvements. An unequal intake-expenditure balance during the training could have induced the unmodified results in fat mass, muscle mass, and body weight. Besides, through nutritional control, the correct recovery could have been guaranteed after each training [93], avoiding the accumulated fatigue. This aspect becomes even more important, considering the age of the participants since this factor could limit their recovery [94]. We did not perform high-end body composition measurements (i.e., computed tomography [CT] or dual-energy X-ray absorptiometry [DXA]), as the authors did not have access to that technology. Hence, greater changes in body composition might have occurred than those, which we were able to determine by the precision of the measurements. Besides, the adaptation of FT-fibers remains an assumption and cannot be proven by high-end body composition measurements. Finally, to estimate the maximum intensity at which the participants could be electrostimulated, a pain threshold test was performed. Pain is a parameter that may have some subjectivity, so we cannot categorically state that the intensity at which the current was applied was that required to cause adaptations.

Practical Applications: Sedentary behavior can be observed mainly in older people, which is a growing population sector. Therefore, to overcome a sedentary lifestyle and prevent the deterioration of their functional fitness, it is necessary to generate physical activity programs with an attractive profile and easily proved benefits. The findings of the present study demonstrate that WB-EMS programs carried out 20 min twice a week under the supervision and guidance of a physical activity technician can be even more effective than only voluntary exercise. Based on the results of this study, to the authors’ opinion, this new training methodology is effective and suitable for postmenopausal women to improve their functional fitness, warranting their independence, decreasing their risk of falls, and improving their quality of life.

Future proposals: The body composition adaptations under the whole body electromyostimulation exposure over a prolonged period and with a greater sample size need to be further researched.

## 5. Conclusions

In summary, the findings suggest that 10 weeks of WB-EMS training with a stimulation frequency of 55 Hz during strength voluntary exercises and 7 Hz during aerobic treadmill walk, in untrained postmenopausal woman, significantly improves power and velocity parameters. However, negligible effects were found in body composition.

## Figures and Tables

**Table 1:** Summary of sample characteristics.

Variable	Total (*n* = 32)	EX + WB-EMS (*n* = 16)	EX (*n* = 16)	*p*-Value
Age (years)	61.38 ± 3.95	63.06 ± 3.42	59.71 ± 3.82	0.011
Body mass (kg)	67.44 ± 10.84	67.11 ± 10.84	67.78 ± 10.12	0.866
Height (cm)	158.32 ± 5.28	156.69 ± 5.02	159.94 ± 5.18	0.081
Body mass index (BMI, kg/m^2^)	26.91 ± 4.11	27.28 ± 4.24	26.54 ± 4.08	0.620

Values are presented as mean ± SD. EX + WB-EMS: exercise plus whole-body electrostimulation group; EX: exercise only group.

**Table 2:** Summary of baseline and postintervention data of the body composition variables for each group EX + WB-EMS (*n* = 16) and EX (*n* = 16).

Variable	Group	Baseline	Post	% Change	MD [95% CI]	*d*
Weight (kg)	EX + WB-EMS	67.11 ± 11.84	66.82 ± 12.10	−0.43	−0.29 [−1.38, 0.80]	−0.19 [−0.68, 0.31]
	EX	67.78 ± 10.12	67.49 ± 10.27	−0.43	−0.28 [−1.38, 0.80]	−0.19 [−0.68, 0.31]
BMI (kg/m^2^)	EX + WB-EMS	27.27 ± 4.24	27.16 ± 4.28	−0.40	−0.11 [−0.56, 0.34]	−0.18 [−0.67, 0.32]
	EX	26.54 ± 4.08	26.46 ± 4.15	−0.30	−0.08 [−0.54, 0.37]	−0.13 [−0.62, 0.36]
Body fat ()	EX + WB-EMS	35.80 ± 5.75	35.99 ± 5.48	0.53	0.19 [−1.13, 1.51]	0.10 [−0.39, 0.59]
	EX	36.61 ± 5.15	35.85 ± 5.39	−2.08	−0.76 [−2.08, 0.56]	−0.41 [−0.91, 0.11]
Fat mass (kg)	EX + WB-EMS	24.56 ± 8.15	24.73 ± 8.38	0.69	0.18 [−2.17, 2.52]	0.05 [−0.44, 0.54]
	EX	26.27 ± 7.79	24.65 ± 7.72	−6.17	−1.61 [−1.96, 0.73]	−0.49 [−100, 0.04]
Lean mass (kg)	EX + WB-EMS	42.57 ± 4.80	42.11 ± 4.37	−1.08	−0.46 [−2.21, 1.30]	−0.18 [−0.68, 0.31]
	EX	41.80 ± 5.42	42.84 ± 3.20	2.49	1.04 [−0.71, 2.80]	0.42 [−0.10, 0.93]
Visceral fat (kg)	EX + WB-EMS	9.06 ± 2.29	9.13 ± 2.36	0.77	0.06 [−2.71, 2.84]	0.02 [−0.47, 0.51]
	EX	10.01 ± 6.07	8.38 ± 2.16	−16.28	−1.64 [−4.41, 1.14]	−0.42 [−0.92, 0.10]
Abdominal fold (mm)	EX + WB-EMS	26.43 ± 9.98	23.44 ± 6.43	−11.31	−2.99 [−6.57, 0.59]	−0.59 [−1.12, −0.05]
	EX	27.85 ± 10.61	27.13 ± 10.86	−2.59	−0.73 [−4.19, 2.74]	−0.15 [−0.64, 0.35]
Waist to hip ratio	EX + WB-EMS	0.83 ± 0.08	0.80 ± 0.06	−3.61	−0.03 [−0.14, 0.09]	−0.16 [−0.65, 0.34]
	EX	0.76 ± 0.22	0.83 ± 0.07	9.21	0.07 [−0.04, 0.18]	0.45 [−0.07, 0.96]
6-fold (mm)	EX + WB-EMS	132.48 ± 35.39	126.06 ± 22.50	−4.85	−6.41 [−19.20, 6.37]	0.35 [−0.85, 0.16]
	EX	130.74 ± 37.62	133.13 ± 37.12	1.83	2.39 [−10.40, 15.17]	0.13 [−0.36, 0.62]
Waist (cm)	EX + WB-EMS	84.13 ± 10.88	83.47 ± 10.11	−0.78	−0.66 [−9.81, 8.48]	−0.05 [−0.54, 0.44]
	EX	79.90 ± 21.64	84.24 ± 11.19	5.43	4.34 [−4.80, 13.48	0.34 [−0.17, 0.83]
Hip (cm)	EX + WB-EMS	102.21 ± 8.28	99.08 ± 14.79	−3.06	−3.14 [−12.22, 5.95]	−0.24 [−0.74, 0.26]
	EX	97.42 ± 14.54	101.37 ± 7.78	4.05	3.95 [−5.13, 13.03]	0.31 [−0.20, 0.80]

Values are presented as mean ± SD. EX + WB-EMS: exercise plus whole-body electrostimulation group; EX: exercise only group; % change: percentage change; MD: mean difference; CI: confidence interval; *d*: Cohen’s *d* effect size; BMI: body mass index.

**Table 3:** Summary of baseline and postintervention data of the mechanical variables of strength exercises for each group EX + WB-EMS (*n* = 16) and EX (*n* = 16).

Variable	Group	Baseline	Post	% Change	MD [95% CI]	*d*
Squat						
Velocity (m·s^−1^)	EX + WB-EMS	0.48 ± 0.10	0,75 ± 0.10	56.25	0.27 [0.20, 0.33] ***	2.85 [1.72, 3.96] ^^^
	EX	0.50 ± 0.05	0.66 ± 0.10	32.00	0.15 [0.09, 0.22] ***	1.63 [0.86, 2.37] ^#^
Power (W)	EX + WB-EMS	478.87 ± 143.32	782.18 ± 194.52	63.34	303.31 [223.18, 383.44] ***	2.67 [1.60, 3.73] ^^^
	EX	548.26 ± 100.43	730.571 ± 160.14	33.25	182.31 [102.18, 262.44] ***	1.61 [0.84, 2.35] ^#^
Bench press						
Velocity (m·s^−1^)	EX + WB-EMS	0.50 ± 0.13	0.81 ± 0.08	62.00	0.39 [0.22, 0.39] ***	2.48 [1.46, 3.47] ^^^
	EX	0.58 ± 0.07	0.59 ± 0.07	1.72	0.01 [−0.08, 0.09]	0.07 [−0.42, 0.56]
Power (W)	EX + WB-EMS	47.17 ± 14.35	78.24 ± 14.02	65.87	31.08 [22.63, 39.51] ***	2.61 [1.55, 3.64] ^^^
	EX	52.45 ± 53.41	53.41 ± 7.00	2.22	0.96 [−7.21, 9.13]	0.08 [−0.41, 0.57]

Values are presented as mean ± SD. EX + WB-EMS: exercise plus whole-body electrostimulation group; EX: exercise only group; % change: percentage change; MD: mean difference; CI: confidence interval; *d*: Cohen’s *d* effect size; *** *p_Bonferroni_* < 0.001; ^#^: Large effect size; ^^^ Very large effect size.

**Table 4:** Summary of the mechanical variables of strength exercises results for each group EX + WB-EMS (*n* = 16) and EX (*n* = 16).

Variable	∆ EX + WB-EMS	∆ EX	MD [95% CI]	*d*
Squat				
Velocity (m·s^−1^)	0.11 ± 0.01	0.06 ± 0.01	0.04 [0.01, 0.08] *	0.98 [0.23, 1.71] ^$^
Power (w)	99.51 ± 11.96	60.83 ± 11.96	38.69 [1.75, 75.62] *	0.81 [0.08, 1.52] ^$^
Bench press				
Velocity (m·s^−1^)	0.13 ± 0.01	0.03 ± 0.01	0.10 [0.06, 0.14] ***	1.90 [1.11, 2.82] ^#^
Power (w)	28.77 ± 2.69	3.12 ± 2.59	25.64 [17.48, 33.82] ***	2.39 [1.49, 3.34] ^^^

Values are presented as estimated mean ± SE. ∆ EX + WB-EMS: change score of exercise plus whole-body electrostimulation group; ∆ EX: change score of exercise only group; MD: mean difference; CI: confidence interval; *d*: Cohen’s *d* effect size; * *p_Bonferroni_* ≤ 0.05; ***: *p_Bonferroni_* < 0.001; ^$^ moderate effect size; ^#^ Large effect size; ^^^ Very large effect size.

## Supplementary Materials

The study data are available online at: https://www.mdpi.com/1660-4601/17/14/4982/s1, Figure S1: Analysis of covariance assessing differences in mechanical variables of strength exercises at the end of follow-up among intervention versus control groups, Table S1: Summary of body composition results for each group EX + WB-EMS (*n* = 16) and EX (*n* = 16).
