# Supplementary material for: Impact of Whole Body Electromyostimulation on Velocity, Power and Body Composition in Postmenopausal Women: A Randomized Controlled Trial
Source: Int J Environ Res Public Health. 2020 Jul 10;17(14):4982. doi: 10.3390/ijerph17144982 (PMC7400631; doi:10.3390/ijerph17144982)
Supplement: Supplementary file 1 [file ijerph-17-04982-s001.pdf]

Table 1. Summary of body composition results for each group EX + WB-EMS (n = 16) and EX (n = 16).

Variable	Δ EX + WB-EMS	Δ EX	MD [95% CI]	*d*
Weight (kg)	-0.44 ± 0.42	-0.15 ± 0.42	-0.29 [-1.60, 1.01]	-0.17, [-0.87, 0.52]
BMI (kg/m^2)	-0.18 ± 0.18	-0.02 ± 0.18	-0.16 [-0.70, 0.38]	-0.22, [-0.92, 0.47]
Body fat (%)	-0.14 ± 0.49	-0.43 ± 0.49	0.29 [-1.22, 1.81]	0.15, [-0.55, 0.84]
Fat mass (kg)	0.54 ± 0.90	-1.97 ± 0.90	2.51 [-0.29, 5.31]	0.70, [-0.02, 1.41]$
Lean mass (kg)	-0.51 ± 0.49	1.10 ± 0.49	-1.61 [-3.15, 0.07]*	-0.82, [-1.54, -0.09]$
Visceral fat (kg)	-0.40 ± 0.56	-1.17 ± 0.56	0.77 [-1.03, 2.57]	0.34, [-0.36, 1.04]
Abdominal fold (mm)	-3.73 ± 1.20	-0.03 ± 1.15	-3.70 [-7.34, -0.06] *	-0.77, [-1.50, -0.06]$
Waist to hip ratio	-0.01 ± 0.02	0.06 ± 0.02	-0.07 [-0.13, -0.02] *	-0.87, [-1.60, -0.14]$
6-fold (mm)	-7.39 ± 4.19	3.36 ± 4.19	-10.75 [-23.67, 2.18]	-0.64, [-1.35, 0.08]$
Waist (cm)	-1.31 ± 2.09	4.99 ± 2.09	-6.30 [-12.84, 0.25]	-0.75, [-1.47, -0.03]$
Hip (cm)	-4.35 ± 2.89	5.16 ± 2.89	-9.51 [-18.66, -0.37] *	-0.82, [-1.54, -0.09]$

Values are presented as estimated mean ± SE. Δ EX + WB-EMS: change score of exercise plus whole-body electrostimulation group; Δ EX: change score of exercise only group; MD: mean difference; CI: confidence interval; *d*: Cohen's *d* effect size; BMI: body mass index; *: $p_{Bonferroni} \leq 0.05$; **: $p_{Bonferroni} < 0.01$; ***: $p_{Bonferroni} < 0.001$; $: moderate effect size.

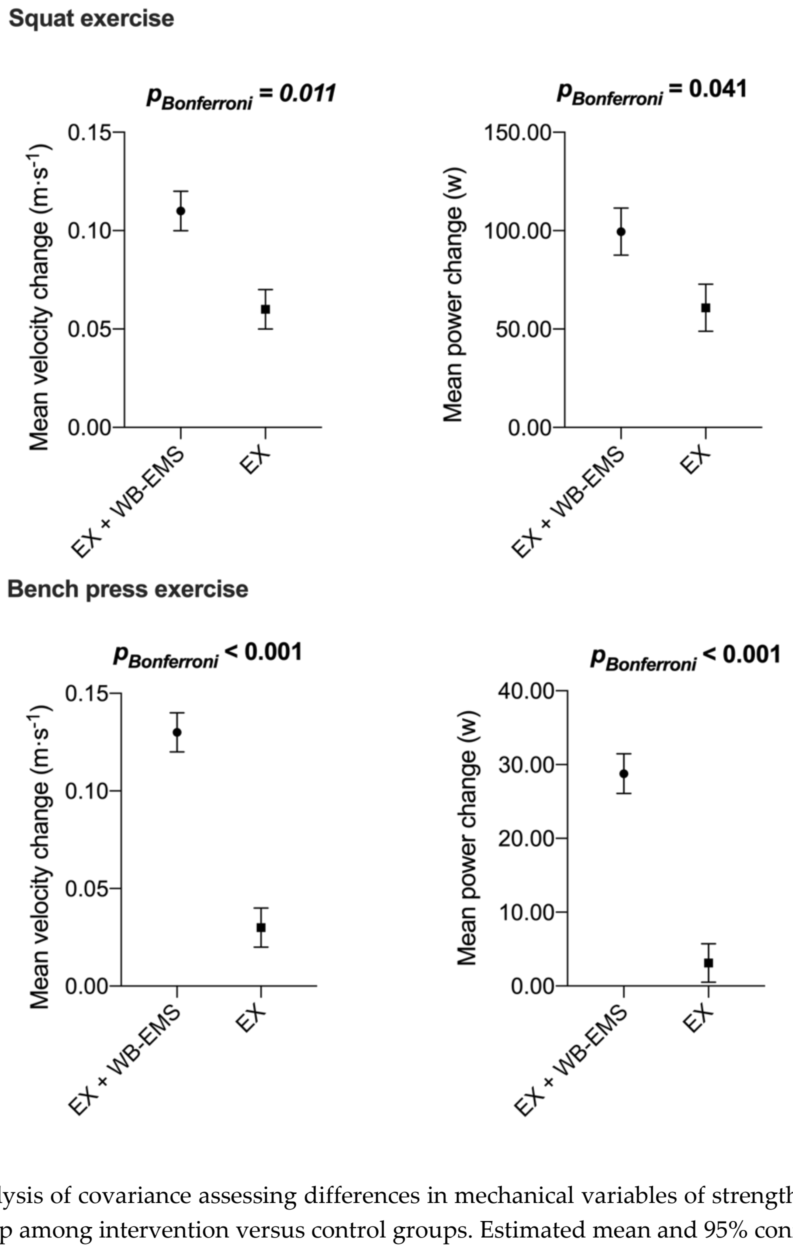

Figure S1. Analysis of covariance assessing differences in mechanical variables of strength exercises at the end of follow-up among intervention versus control groups. Estimated mean and 95% confidence intervals

(CIs; error bars) represent values after adjusting by age and the corresponding value of the mechanical variable at baseline. Statistically significant values are shown in bold.
